# Simultaneous monitoring of static and dynamic intracranial pressure parameters from two separate sensors in patients with cerebral bleeds: comparison of findings

**DOI:** 10.1186/1475-925X-11-66

**Published:** 2012-09-07

**Authors:** Per Kristian Eide, Sverre Holm, Wilhelm Sorteberg

**Affiliations:** 1 Department of Neurosurgery, Oslo University Hospital, Rikshospitalet, Oslo, Norway; 2 Faculty of Medicine, University of Oslo, Oslo, Norway; 3 Department of Informatics, University of Oslo, Oslo, Norway

## Abstract

**Background:**

We recently reported that in an experimental setting the zero pressure level of solid intracranial pressure (ICP) sensors can be altered by electrostatics discharges. Changes in the zero pressure level would alter the ICP level (mean ICP); whether spontaneous changes in mean ICP happen in clinical settings is not known. This can be addressed by comparing the ICP parameters level and waveform of simultaneous ICP signals. To this end, we retrieved our recordings in patients with cerebral bleeds wherein the ICP had been recorded simultaneously from two different sensors. Materials and Methods: During a time period of 10 years, 17 patients with cerebral bleeds were monitored with two ICP sensors simultaneously; sensor 1 was always a solid sensor while Sensor 2 was a solid -, a fluid - or an air-pouch sensor. The simultaneous signals were analyzed with automatic identification of the cardiac induced ICP waves. The output was determined in consecutive 6-s time windows, both with regard to the static parameter mean ICP and the dynamic parameters (mean wave amplitude, MWA, and mean wave rise time, MWRT). Differences in mean ICP, MWA and MWRT between the two sensors were determined. Transfer functions between the sensors were determined to evaluate how sensors reproduce the ICP waveform.

**Results:**

Comparing findings in two solid sensors disclosed major differences in mean ICP in 2 of 5 patients (40%), despite marginal differences in MWA, MWRT, and linear phase magnitude and phase. Qualitative assessment of trend plots of mean ICP and MWA revealed shifts and drifts of mean ICP in the clinical setting. The transfer function analysis comparing the solid sensor with either the fluid or air-pouch sensors revealed more variable transfer function magnitude and greater differences in the ICP waveform derived indices.

**Conclusions:**

Simultaneous monitoring of ICP using two solid sensors may show marked differences in static ICP but close to identity in dynamic ICP waveforms. This indicates that shifts in ICP baseline pressure (sensor zero level) occur clinically; trend plots of the ICP parameters also confirm this. Solid sensors are superior to fluid – and air pouch sensors when evaluating the dynamic ICP parameters.

## Background

Monitoring of the intracranial pressure (ICP) is crucial in the management of neurosurgical patients [1-3]. The goal is then usually to keep the static pressure parameter mean ICP or the dynamic pressure parameter mean ICP wave amplitude (MWA) below certain threshold levels; i.e. the mean ICP <20-25 mmHg [1,2], or the MWA <5 mmHg [3].

The ICP is most often measured using a solid sensor or through a fluid catheter placed within a cerebral ventricle [4]. Though today’s practice of ICP monitoring spans decades [5], newer data have shown that the level of the ICP (i.e. the static ICP or the mean ICP) can be sensitive to inherent weaknesses in the sensors. We hence recently reported that in an experimental setting spontaneous shifts in the baseline pressure (sensor zero pressure) of a solid sensor can be triggered by electrostatic discharges (ESDs) [6]. From this, one may ask if this also happens in clinical settings, and whether the ICP scores that are presented on the monitoring screen represent reality or not.

The questions raised above may be addressed by comparing simultaneous signals obtained from two separate sensors placed intracranially in the same patient. To this end, we retrieved our ICP recordings in all 17 patients with cerebral bleeds wherein the ICP had been monitored simultaneously from two separate sensors. We then compared the mean ICP (static pressure parameter) as well as the mean wave amplitude (MWA) and mean wave rise time (MWRT) (dynamic pressure parameters) of the two sensors. With special emphasis on the quality of the dynamic ICP signals, the transfer function of the sensor types used in the 17 patients were also assessed.

## Methods

### Patient recordings

The ICP recordings were retrieved from patients managed for aneurysmal subarachnoid haemorrhage (SAH) and/or intra-cerebral haemorrhage (ICH) at the Department of Neurosurgery, Oslo University Hospital – Rikshospitalet during the time period 2002–2011. Only patients wherein management included simultaneous monitoring from two separate ICP sensors were included.

The Regional Committee for Medical and Health Research Ethics (REK) of Health Region South-East, Norway was informed in writing, and had no objections to the study. The study was approved by the Oslo University Hospital – Rikshospitalet as a quality study.

### ICP monitoring and analysis

The setup for the simultaneous ICP monitoring was as follows: Sensor 1 was always a solid (strain-gauge) sensor (Codman Microsensor, Codman MicroSensor, Johnson and Johnson, Raynham, Massachusetts, USA), while Sensor 2 was either (a) another solid sensor (Codman Microsensor, Codman MicroSensor, Johnson and Johnson, Raynham, Massachusetts, USA; Category A), (b) a fluid sensor (Edward’s fluid sensor) connected to an external ventricular drain (Truwave PX-600 F Pressure Monitoring Set, Edwards Life sciences LLC, Irvine, CA, USA; Category B), or (c) an air-pouch sensor (Spiegelberg intraparenchymal probe 3PN, Spiegelberg KG, Hamburg, GE; Category C). Both ICP sensors were implanted at the same time.

The ICP sensors were introduced to the intracranial compartment either via a small burr hole and a minimal opening in the dura or via the craniotomy used for aneurysm clipping/hematoma evacuation. The solid sensor was placed within the brain parenchyma, and connected via cable to the ICP Express (Codman ICP Express, Johnson and Johnson, Raynham, Massachusetts, USA). The fluid sensor was connected outside the patient to an external ventricular drain (EVD) that had been placed in the ventricular fluid, while the air-pouch sensor was placed inside the brain parenchyma, and connected to a Spiegelberg ICP Monitor (Spiegelberg KG, Hamburg, Ge). The ICP signals from all sensors were passed to a vital signs Siemens 9000 XL Series Monitor (Siemens Medical Systems Inc., Danvers, MA, USA). By means of the Siemens Infinity Gateway Software (Siemens Medical Systems Inc., Danvers, MA, USA), the continuous ICP signals were transferred online via the hospital network to a computer server and stored as raw data files (sampling rate 100 Hz).

The analysis of the continuous ICP waveforms was done using a previously published method for automatic cardiac induced ICP waves [7] that has been implemented in the software (Sensometrics Software, dPCom As, Oslo). In short, the process is as follows: (1) From the continuous pressure signal, each pressure wave is identified by its beginning and ending diastolic minimum pressure, and its systolic maximum pressure. (2) For each pressure wave a set of single wave parameters is determined such as rise time (dT), amplitude (dP) and rise time coefficient (RT). (3) Each pressure wave is differentiated as either a cardiac-beat-induced ICP wave, or as an artefact-induced wave, depending on whether the single wave parameters meet defined requirements. Thus, cardiac induced waves have single wave parameters (e.g. dT, dP, RT) within defined threshold values 3) Then, the identified cardiac-induced waves are applied for further analysis; analysis is done during time windows of 6-s duration containing. Only 6-s time windows containing minimum 4 cardiac beat induced waves were considered to be of good quality, and were used for the present analysis. Thus, for each 6-s time window the mean ICP and the ICP waveform indices mean wave amplitude (MWA) and mean wave rise time (MWRT) were determined. For a 6-s time window to be accepted according to the automatic method, it contained minimum four cardiac induced waves. Accordingly, this method automatically differentiates between pressure waves induced by the cardiac contractions and artefact waves due to noise in the pressure signal (e.g. due to patient movement, or sensor movement or dysfunction); artefact waves were hence omitted from the analysis.

**Figure 1:**
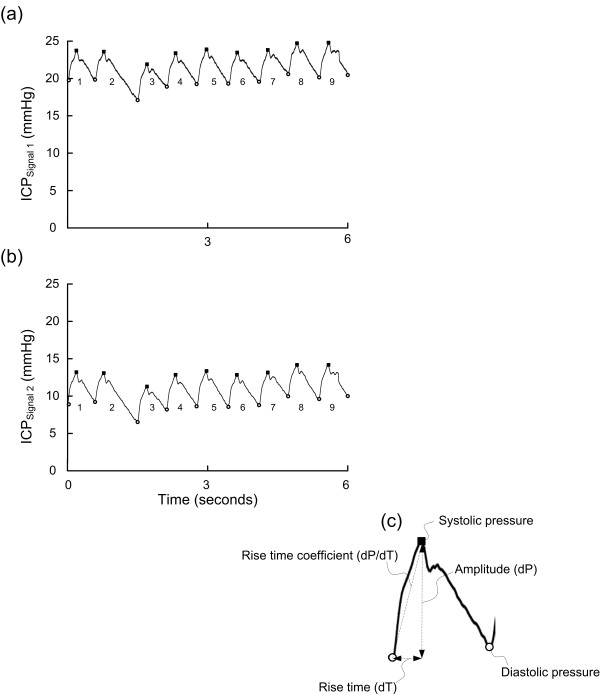
**Simultaneous 6-s time windows of Signals 1 and 2 (PatID 2).** A 6-s time window of the raw signal of (**a**) Signal 1 and (**b**) Signal 2 of PatID 2, showing the cardiac-induced ICP waves. For every cardiac-induced wave the amplitude (dP) and rise time (dT) were automatically determined (**c**). Mean ICP, mean ICP wave amplitude (MWA) and rise time (MWRT) were determined for each 6-s time window; for the 6-s time window shown in a-b, mean ICP was 21.3 mmHg (Signal 1) and 10.9 mmHg (Signal 2), MWA was 4.1 mmHg (Signal 1) and 4.1 mmHg (Signal 2), and MWRT was 0.21 sec (Signal 1) and 0.20 sec (Signal 2).

For this particular study, intracranial pressure recordings from the corresponding 6-s time windows containing simultaneous ICP signals and with identical time references were then compared. A 6-s time window with two ICP signals (PatID 2) is shown in Figure 1.

For each signal in every recording we determined the average values of mean ICP, MWA and MWRT during the whole observation period as well as the percentage of 6-s time windows, with differences between signals of mean ICP >5 mmHg or >10 mmHg, MWA >1 mmHg or >2 mmHg, and MWRT >0.1 sec or >0.2 sec, respectively.

### Visual inspection of trend plots of mean ICP and MWA

For the 17 patient recordings, the trend plots of mean ICP and MWA for simultaneous recordings were inspected for the occurrence of shifts or drifts of mean ICP relative to MWA. This was done to qualitatively assess whether shifts in mean ICP occurred in the clinical setting.

### Assessment of transfer function for the different types of sensors

**Figure 2:**
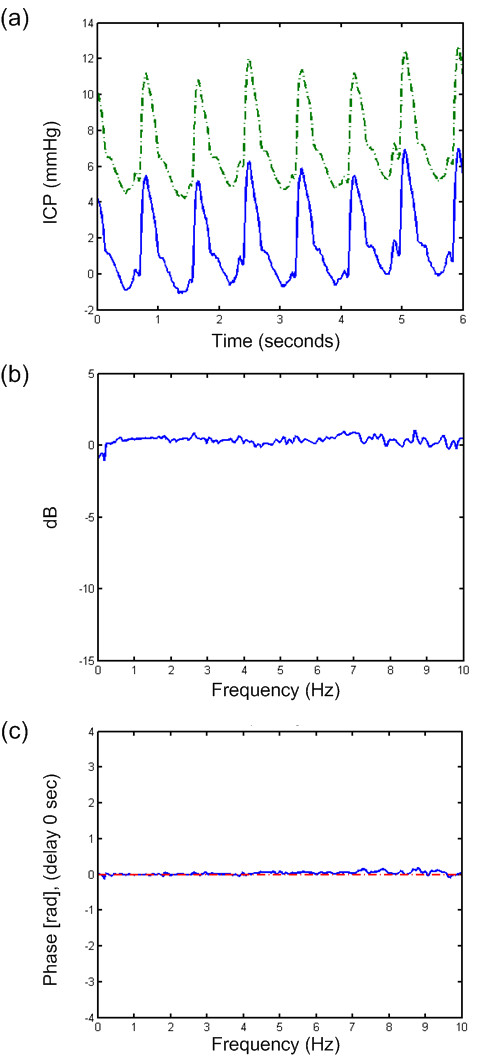
**Transfer function of a 6-s time window of PatID 3.** Analysis of one 6-s time window of PatID 3 showing (**a**) signals 1 (Codman) and 2 (dash-dot line, Codman), (**b**) transfer function of magnitude, and (**c**) transfer function of phase with dash-dot line indicating estimate based on a delay of 0 sec. In this case, 15.6% of 6-s time windows showed a difference in MWA >1 mmHg (largest MWA in Signal 1 in 11.3% and largest MWA in Signal 2 in 4.3%). In 0.5% of 6-s time windows, the difference in MWRT was >0.1 sec (largest MWRT in Signal 1 in 0.2% and largest MWRT in Signal 2 in 0.3%).

**Figure 3:**
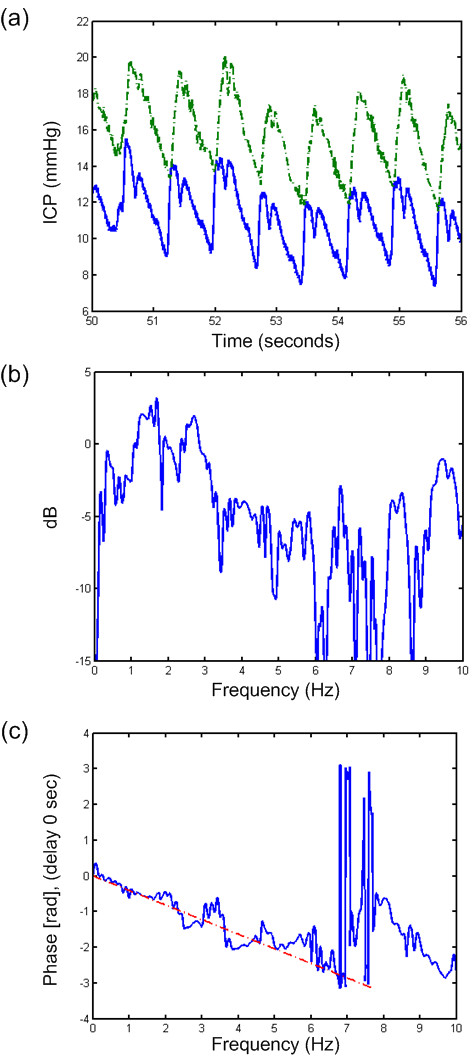
**Transfer function of a 6-s time window of PatID 6.** Analysis of one 6-s time window of PatID 6 showing (**a**) the signals 1(Codman) and 2 (dash-dot line, Edwards), (**b**) transfer function of magnitude, and (**c**) transfer function of phase with dash-dot line indicating estimate based on a delay of 0.065 sec. In this case, 47.1% of 6-s time windows showed a difference in MWA >1 mmHg (largest MWA in Signal 1 in 18.9% and largest MWA in Signal 2 in 28.2%). In 12.8% of 6-s time windows, the difference in MWRT was >0.1 sec (largest MWRT in Signal 1 in 12.7% and largest MWRT in Signal 2 in 0.1%).

**Figure 4:**
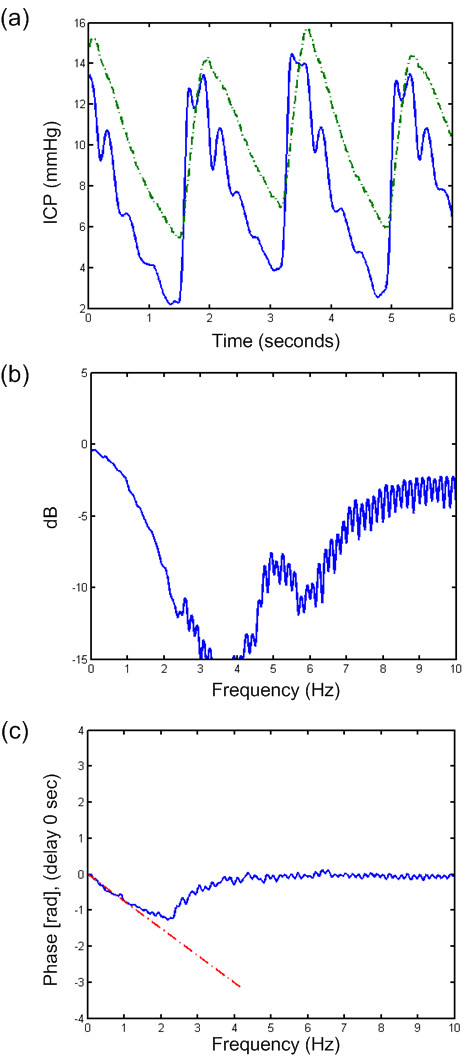
**Transfer function of a 6-s time window of PatID 15.** Analysis of one 6-s time window of PatID 15 showing (**a**) signal 1 (Codman) and 2 (dash-dot line, Spiegelberg), (**b**) transfer function of magnitude, and (**c**) transfer function of phase with dash-dot line indicating estimate based on a delay of 0.12 sec. In this case, 80.4% of 6-s time windows showed a difference in MWA >1 mmHg (largest MWA in Signal 1 in 51.6% and largest MWA in Signal 2 in 28.8%). In 7.6% of 6-s time windows, the difference in MWRT was >0.1 sec (largest MWRT in Signal 1 in 3.2% and largest MWRT in Signal 2 in 4.4%).

The transfer function was estimated using the Matlab function tfestimate.m (Mathworks®, version R2011a with Signal Processing Toolbox version 6.15). It estimates the linear filter required to transform the second transducer’s data (Signal 2) into that of the reference transducer (Codman; Signal 1). The transfer function is the quotient of the cross power spectral density (*P*_*21*_) of signals 1 and 2 and the power spectral density (*P*_*11*_) of signal 1, T_21_(f) = P_21_(f)/P_11_(f). The transfer function has a magnitude and a phase as indicated in Figures 2, 3,4 b and c. It has been estimated by averaging over Fourier transformed 6-s time windows, spanning from a minimum of 36 seconds (6 segments). Because the mean value was subtracted for each time window, the transfer function analysis is not influenced by variations in mean ICP. The phase corresponds to the delay, τ, between the two signals according to φ = −2πfτ. It has been checked by estimating the delay in the time domain. This can be done by inspecting the time plots (Figures 2,3 and 4a), but a more robust measure can be found by estimating the cross-correlation function (Matlab xcorr.m). The phase is a linear function which has been plotted with red dotted lines on top of the transfer function phase. In all of the transfer-function plots it matches very well with the estimated phase.

## Results

### Patient recordings

Table 1 gives age, gender and type of bleed in the 17 patients included in the study, while Table 2 shows type and location of the ICP sensors and number of accepted (good quality) 6-s time windows containing two signals. For all patients combined, a total of 441,654 6-s time windows were analyzed (Table 2).

**Table 1:** Demographic data of 17 patients with cerebral bleeds

**PatID**	**Age**	**Gender**	**Type of bleed**
**Category A**			
1	66	M	SAH (ACOM)
2	76	M	ICH (right parieto-occipital)
3	39	F	SAH (ACOM)
4	72	F	SAH (left MCA)
5	59	F	SAH (left MCA)
**Category B**			
6	56	M	SAH (BA)
7	48	M	SAH (left MCA)
8	60	M	SAH (ACOM)
9	50	F	SAH (right VA)
10	55	F	SAH (ACOM)
**Category C**			
11	66	M	ICH (right frontal)/IVH
12	56	F	SAH (right MCA)
13	60	F	SAH (BA/left ICA)
14	54	M	SAH (left PCOM)
15	67	M	SAH (right PCOM)
16	71	M	ICH (cerebellum)
17	82	F	ICH (cerebellum)

ACOM: anterior communicating artery; BA: basilar artery; ICA: internal carotid artery;

ICH: Intra-cerebral haemorrhage; IVH: intra-ventricular haematoma; MCA: middle cerebral

artery; PCOM: posterior communicating artery; SAH: Subarachnoid haemorrhage;

VA: vertebral artery.

**Table 2:** Sensor type, sensor location and number of accepted simultaneous 6-s time windows

**PatID**	**Sensor type**		**Sensor location**		**Number of simultaneous 6-s time windows**
	**Signal 1**	**Signal 2**	**Signal 1**	**Signal 2**	
**Category A**					
1	Solid	Solid	Left frontal lobe	Right frontal lobe	6,149
2	_“_	_“_	Left frontallobe	Left occipital lobe	31,488
3	_“_	_“_	Right frontal lobe	Right occipital lobe	8,208
4	_“_	_“_	Left frontal lobe	Left frontal ventricular horn	21,435
5	_“_	_“_	Right frontal lobe	Right frontal ventricular horn	19,703
**Category B**					
6	Solid	Fluid	Right frontal lobe	Right frontal ventricular horn	6,295
7	_“_	_“_	Right frontal lobe	Right frontal ventricular horn	26,677
8	_“_	_“_	Right frontal lobe	Right frontal ventricular horn	4,489
9	_“_	_“_	Right frontal lobe	Right frontal ventricular horn	2,555
10	_“_	_“_	Left frontal lobe	Left frontal ventricular horn	6,262
**Category C**					
11	Solid	Air-pouch	Right frontal lobe	Right frontal ventricular horn	44,445
12	_“_	_“_	Right frontal lobe	Right frontal ventricular horn	93,506
13	_“_	_“_	Right frontal lobe	Right frontal ventricular horn	52,137
14	_“_	_“_	Right frontal lobe	Right frontal ventricular horn	37,192
15	_“_	_“_	Left frontal lobe	Left frontal ventricular horn	38,274
16	_“_	_“_	Right frontal lobe	Right frontal ventricular horn	588
17	_“_	_“_	Right frontal lobe	Right frontal ventricular horn	42,251

### Comparisons of ICP parameters between simultaneous signals

#### Mean ICP

Table 3, left presents the mean ICP recorded by the two sensors. Differences in mean ICP >5 mmHg in >20% of observations were seen in 2 of 5 patients (40%) when using two solid sensors (Additional file 1: Category A; Table 3, right), in 4 of 5 patients (80%) when using one solid and one fluid sensor (Additional file 2: Category B), and in 1of 7 patients (14%) when using one solid and one air-pouch sensor (Additional file 3: Category C).

**Table 3:** Differences in mean ICP between simultaneous ICP signals

**PatID**	**Mean ICP (mmHg; average + std)**		**Percentage of 6-s time windows with difference in mean ICP:**	
	**Signal 1**	**Signal 2**	**>5 mmHg**	**>10 mmHg**
**Category A**				
1	5.3 + 2.7	8.1 + 2.3	2	-
2	9.2 + 2.9	18.1 + 3.6	100	50
3	9.3 + 3.7	9.5 + 3.0	11	2
4	8.1 + 2.9	2.7 + 2.3	68	-
5	9.1 + 2.3	5.8 + 2.5	-	-
**Category B**				
6	15.3 + 3.4	20.3 + 2.3	37	2
7	9.3 + 4.6	7.6 + 5.5	35	4
8	6.9 + 4.1	9.5 + 5.3	37	-
9	−0.48 + 2.4	−29.6 + 4.6	100	100
10	11.9 + 5.4	12.5 + 5.2	5	-
**Category C**				
11	11.4 + 2.6	14.8 + 2.3	4	3
12	9.3 + 3.5	9.5 + 3.4	1	1
13	10.0 + 3.6	9.0 + 5.1	19	6
14	10.3 + 2.8	5.9 + 6.5	14	9
15	7.8 + 14.3	8.5 + 4.1	27	6
16	14.1 + 1.9	10.6 + 3.7	14	1
17	15.8 + 5.5	15.5 + 3.8	8	5

#### Mean ICP wave amplitude (MWA)

Table 4, gives the MWA recorded by the two sensors and percentage of simultaneous 6-s time windows where the MWA of the two sensors differed >1 mm Hg and >2 mm Hg, respectively. Differences were marginal when using two solid sensors. (Additional file 1: Category A). The patient with two solid sensors that had differences in >1 mmHg in 16% of the 6-s time windows (PatID 3), were one out of two patients where one sensor had been placed frontal and the other occipital. The other patient with such sensor placements (PatID 2), presented with considerable differences in mean ICP but with similar MWAs.

**Table 4:** Differences in mean wave amplitude (MWA) between simultaneous ICP signals

**PatID**	**MWA (mmHg; average + std)**		**Percentage of 6-s time windows with difference in MWA:**	
	**Signal 1**	**Signal 2**	**>1 mmHg**	**>2 mmHg**
Category A				
1	4.5 + 0.7	4.4 + 0.7	2	-
2	4.5 + 0.7	4.5 + 0.7	1	-
3	6.8 + 1.2	7.0 + 1.2	16	-
4	3.2 + 0.5	3.1 + 0.5	-	-
5	1.9 + 0.8	1.6 + 0.6	-	-
Category B				
6	6.4 + 0.9	6.5 + 1.2	47	10
7	4.5 + 1.2	4.3 + 1.3	23	7
8	8.9 + 3.2	8.9 + 4.1	59	21
9	3.0 + 0.3	2.4 + 0.6	19	-
10	7.1 + 3.1	6.6 + 2.9	27	9
Category C				
11	7.2 + 1.0	6.2 + 0.9	52	4
12	5.6 + 1.8	4.9 + 1.3	41	18
13	4.4 + 1.4	3.9 + 1.7	29	2
14	6.7 + 1.7	5.2 + 3.2	90	65
15	7.6 + 14.3	7.1 + 14.3	80	64
16	9.3 + 0.5	8.3 + 0.6	30	1
17	8.0 + 2.1	7.3 + 2.3	38	6

MWA: mean wave amplitude.

A difference in MWA >1 mmHg in more than 20% of the observations was seen in 4/5 (80%) of patients when using one solid and one fluid sensor (Additional file 2: Category B), and in all 7 patients (100%) when using one solid and one air-pouch sensor (Additional file 3: Category C; Table 4).

#### Mean ICP wave rise time (MWRT)

Table 5, presents MWRT as well as percentage of simultaneous 6-s time windows where the MWRT of the two sensors differed >0.1 s and >0.2 s, respectively. Marginal differences in MWRT were seen when comparing two solid sensors were seen (Category A). Also, when comparing one solid and one fluid sensor (Category B), differences were small. In contrast, when comparing one solid and one air-pouch sensor, differences were >0.1 s in more than 20% of observations in 3 of 7 patient recordings (43%; Category C).

**Table 5:** Differences in mean wave rise time (MWRT) between simultaneous ICP signals

**PatID**	**MWRT (sec; average + std)**		**Percentage of 6-s time windows with difference in MWRT:**	
	**Signal 1**	**Signal 2**	**>0.1 s**	**>0.2 s**
**Category A**				
**1**	0.27 + 0.04	0.27 + 0.04	-	-
**2**	0.21 + 0.02	0.21 + 0.02	-	-
**3**	0.14 + 0.04	0.14 + 0.03	1	-
**4**	0.18 + 0.05	0.18 + 0.05	-	-
**5**	0.28 + 0.06	0.28 + 0.05	-	-
**Category B**				
**6**	0.29 + 0.04	0.24 + 0.04	13	-
**7**	0.25 + 0.02	0.25 + 0.04	2	-
**8**	0.23 + 0.02	0.21 + 0.03	2	-
**9**	0.14 + 0.04	0.14 + 0.04	3	1
**10**	0.28 + 0.03	0.24 + 0.06	14	-
**Category C**				
**11**	0.26 + 0.05	0.28 + 0.02	9	-
**12**	0.33 + 0.13	0.23 + 0.09	45	17
**13**	0.36 + 0.06	0.45 + 0.12	50	20
**14**	0.33 + 0.09	0.39 + 0.10	25	7
**15**	0.22 + 14.3	0.22 + 14.3	8	1
**16**	0.12 + 0.01	0.12 + 0.01	-	-
**17**	0.24 + 0.05	0.25 + 0.03	7	-

MWRT: mean wave rise time.

### Examples of trend plots of mean ICPs and MWAs

**Figure 5:**
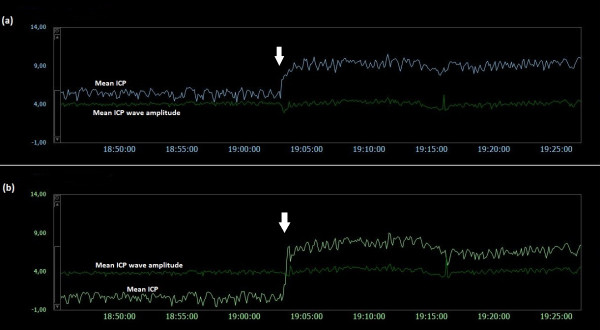
**Trend plots of mean ICP/mean ICP wave amplitude of Signals 1 and 2 (PatID 1).** The trend plots of mean ICP and mean ICP wave amplitude (MWA) of (**a**) Signal 1 and (**b**) Signal 2 of PatID 1 is shown [Average of mean ICP: 7.6 mmHg (Signal 1), 4.3 mmHg (Signal 2). Average of mean ICP wave amplitude: 4.1 mmHg (Signal 1), 4.0 mmHg (Signal 2)]. The vertical arrows indicate sudden changes in mean ICP without accompanying changes in MWA; mean ICP changed differently for the two signals, being more extensive for Signal 2.

**Figure 6:**
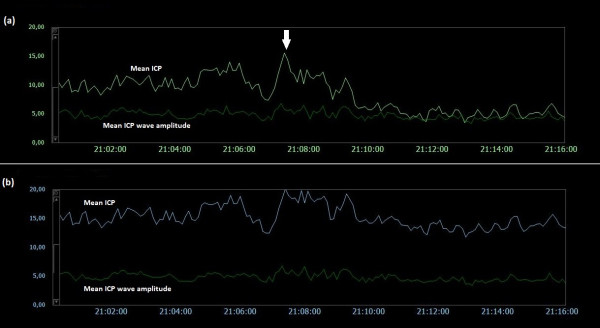
**Trend plots of mean ICP/mean ICP wave amplitude of Signals 1 and 2 (PatID 2).** The trend plots of mean ICP and mean ICP wave amplitude (MWA) of (**a**) Signal 1 and (**b**) Signal 2 of PatID 2 is shown [Average of mean ICP: 8.4 mmHg (Signal 1), 15.2 mmHg (Signal 2). Average of mean ICP wave amplitude: 4.8 mmHg (Signal 1), 4.8 mmHg (Signal 2)]. The vertical arrow in (**a**) indicates a drift in mean ICP without accompanying change in MWA. While mean ICP drifted in (**a**), no such simultaneous change in mean ICP in (**b**) was seen.

**Figure 7:**
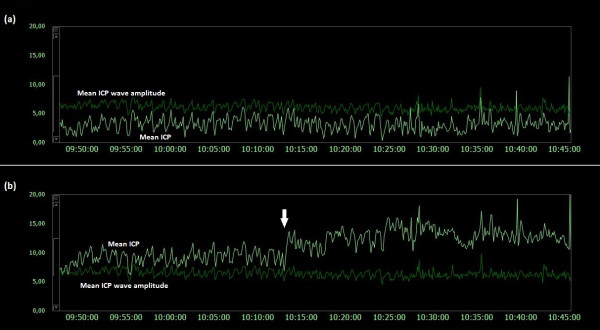
**Trend plots of mean ICP/mean ICP wave amplitude of Signals 1 and 2 (PatID 3).** The trend plots of mean ICP and mean ICP wave amplitude (MWA) of (**a**) Signal 1 and (**b**) Signal 2 of PatID 3 is shown [Average of mean ICP: 3.2 mmHg (Signal 1), 11.3 mmHg (Signal 2). Average of mean ICP wave amplitude: 6.1 mmHg (Signal 1), 6.4 mmHg (Signal 2)]. The vertical arrow in (**b**) indicates a shift in mean ICP without accompanying changes in MWA; no accompanying change in mean ICP in (**a**) was seen.

**Figure 8:**
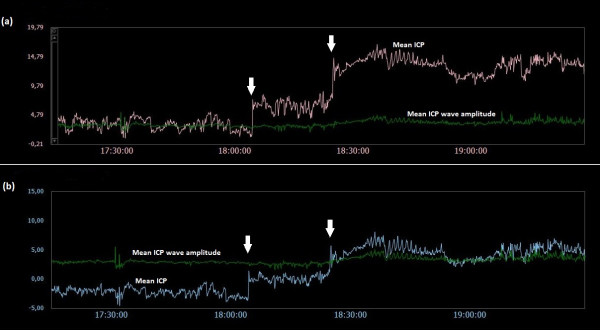
**Trend plots of mean ICP/mean ICP wave amplitude of Signals 1 and 2 (PatID 4).** The trend plots of mean ICP and mean ICP wave amplitude (MWA) of (**a**) Signal 1 and (**b**) Signal 2 of PatID 4 is shown [Average of mean ICP: 8.6 mmHg (Signal 1), 1.5 mmHg (Signal 2). Average of mean ICP wave amplitude: 3.3 mmHg (Signal 1), 3.2 mmHg (Signal 2)]. The vertical arrows in (**a**) and (**b**) indicate shifts in mean ICP without accompanying changes in MWA; mean ICP shifted differently for the two signals, being more extensive for Signal 1.

**Figure 9:**
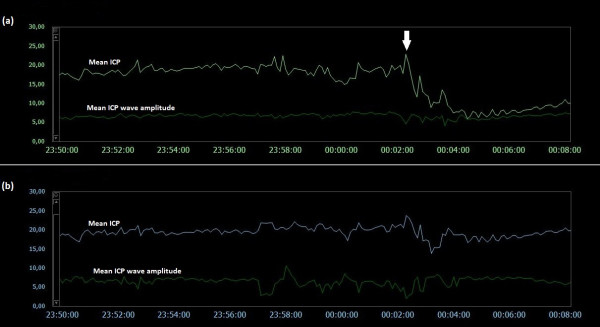
**Trend plots of mean ICP/mean ICP wave amplitude of Signals 1 and 2 (PatID 6).** The trend plots of mean ICP and mean ICP wave amplitude (MWA) of (**a**) Signal 1 and (**b**) Signal 2 of PatID 6 is shown [Average of mean ICP: 15.7 mmHg (Signal 1), 19.5 mmHg (Signal 2). Average of mean ICP wave amplitude: 6.7 mmHg (Signal 1), 6.7 mmHg (Signal 2)]. The vertical arrow in (**a**) indicates a shift in mean ICP without accompanying change in MWA. The shift of mean ICP in (**a**) was not accompanied by a simultaneous shift in mean ICP in (**b**).

**Figure 10:**
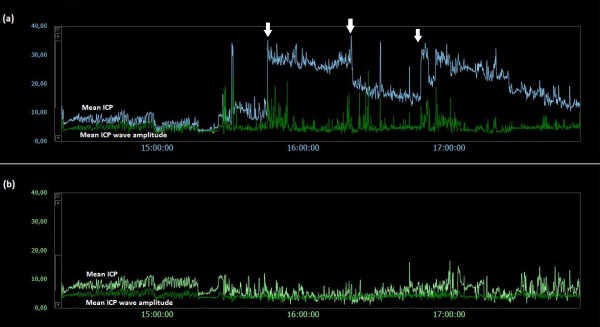
**Trend plots of mean ICP/mean ICP wave amplitude of Signals 1 and 2 (PatID 7).** The trend plots of mean ICP and mean ICP wave amplitude (MWA) of (**a**) Signal 1 and (**b**) Signal 2 of PatID 7 is shown [Average of mean ICP: 16.3 mmHg (Signal 1), 6.4 mmHg (Signal 2). Average of mean ICP wave amplitude: 4.5 mmHg (Signal 1), 4.2 mmHg (Signal 2)]. The vertical arrows in (**a**) indicate shifts in mean ICP without accompanying changes in MWA. The shifts of mean ICP in (**a**) were not accompanied by simultaneous shifts in mean ICP in (**b**).

**Figure 11:**
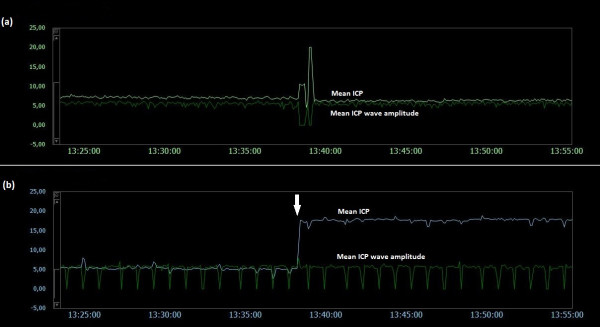
**Trend plots of mean ICP/mean ICP wave amplitude of Signals 1 and 2 (PatID 12).** The trend plots of mean ICP and mean ICP wave amplitude (MWA) of (**a**) Signal 1 and (**b**) Signal 2 of PatID 12 is shown [Average of mean ICP: 6.8 mmHg (Signal 1), 11.9 mmHg (Signal 2). Average of mean ICP wave amplitude: 5.6 mmHg (Signal 1), 5.7 mmHg (Signal 2)]. The vertical arrow in (**b**) indicates a shift in mean ICP without accompanying change in MWA. The shift of mean ICP in (**b**) was not accompanied by a simultaneous shift in mean ICP in (**a**).

**Figure 12:**
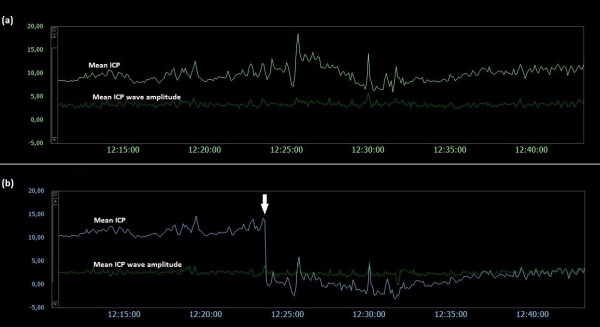
**Trend plots of mean ICP/mean ICP wave amplitude of Signals 1 and 2 (PatID 13).** The trend plots of mean ICP and mean ICP wave amplitude (MWA) of (**a**) Signal 1 and (**b**) Signal 2 of PatID 13 is shown [Average of mean ICP: 9.8 mmHg (Signal 1), 5.0 mmHg (Signal 2). Average of mean ICP wave amplitude: 3.4 mmHg (Signal 1), 2.5 mmHg (Signal 2)]. The vertical arrow in (**b**) indicates a shift in mean ICP without accompanying change in MWA. The shift of mean ICP in (**b**) was not accompanied by a simultaneous shift of mean ICP in (**a**).

**Figure 13:**
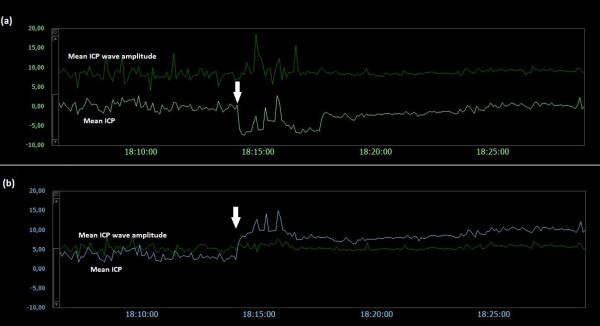
**Trend plots of mean ICP/mean ICP wave amplitude of Signals 1 and 2 (PatID 15).** The trend plots of mean ICP and mean ICP wave amplitude (MWA) of (**a**) Signal 1 and (**b**) Signal 2 of PatID 15 is shown [Average of mean ICP: -1.3 mmHg (Signal 1), 7.2 mmHg (Signal 2). Average of mean ICP wave amplitude: 8.7 mmHg (Signal 1), 5.5 mmHg (Signal 2)]. The vertical arrows in (**a**) and (**b**) indicate shifts in mean ICP; no apparent lasting changes in MWA were seen. The shifts of mean ICP in (**a**) and (**b**) were in opposite directions.

Figures 5,6,7,8,9,10,11,12 and 13 show trend plots of mean ICPs and MWAs in 9 different patients. Figures 5-8 are from Category A patients (PatID´s 1–4) and compared findings in two solid sensors. The figures demonstrate shifts and drifts in mean ICP of both sensors, but with different profiles of shifts between the two simultaneous signals. Figures 9-10 are from Category B patients (PatID´s 9–10), comparing one solid and one fluid sensor. The figures reveal shifts in mean ICP of the solid sensor, but with no accompanying shifts in the fluid sensor. Finally, Figures 11-13 present findings in Category C Patients (PatID´s 11, 13 and 15), comparing one solid and one air pouch sensor. Shifts in mean ICP of the air-pouch sensor were found in both PatID’s 12 and 13. In PatID 15 the shifts of mean ICP occurred in opposite directions for the solid and the air-pouch sensor.

### Impact of sensor characteristics on ICP waveform reproduction

While comparison of two solid sensors disclosed flat transfer function magnitude (Figure 2b) and no phase delay (lower panel), Figure 2 (Figure 2c), comparing one solid and one fluid sensor indicated some delay in the fluid sensor (Figure 3c). The delay was even larger when comparing one solid and one air-pouch sensor (Figure 4c). In the example retrieved from PatID 6 (Figure 3), the heart rate was about 75 beats per minute (1.3 Hz), at which frequency the transfer function was positive (Figure 3b). The phase was linear up to 5–6 Hz (Figure 3c). The positive gain at the frequency of the heart beat is consistent with a larger estimate for the MWA for the Edwards sensor. This was the case in the majority of the 6-second segments with different MWA-values. Of the 47% of segments where the MWA values differed more than 1 mmHg, 28.2% of all segments were for this case. When comparing one solid and one air-pouch sensor, the transfer function falls off for frequencies above 60–120 heart beats per minute (1–2 Hz) (Figure 4b). In this subject (PatID 15), the delay was linear in the same limited area. The heart rate was about 40 beats per minute (0.63 Hz), and the air pouch sensor’s gain was about 2 dB below that of the solid sensor. This is consistent with the statistical data for this subject since for the 80% of time windows where the MWA differed more than 1 mmHg, the majority (51.6% of all time windows) the MWA was larger for the Codman sensor. In this case the transfer function showed a clear low pass character, but in other cases this was not the case and varied much more over the interesting frequency interval up to 5–6 Hz.

## Discussion

The main findings of the present study were that when comparing solid ICP sensors, major differences in ICP level despite close to identical ICP waveform occurred in 2 of 5 (40%) of patients. Inspection of the trend plots of static and dynamic pressure parameters showed that shifts in mean ICP (due to spontaneously changed ICP baseline pressure) occur clinically, most often in the solid sensors. The ICP waveform is reproduced with variable quality by the fluid-filled and air pouch sensors.

The ICP sensors applied in this study are widely used. The solid Codman sensor [8-12] was introduced in the 1980’s while the air-pouch Spiegelberg ICP sensor [13,14] has been used since the 1990’s. The fluid Edward’s sensor has been used extensively in monitoring of various fluid-pressures such as arterial blood pressure, intraventricular pressure and central venous pressure. All of these technologies represent state-of-the art ICP monitoring.

### ICP parameters of the study

Both static and dynamic intracranial pressure parameters were registered and data are presented as average values for the whole observation period and as well as percentage of differences between simultaneous 6-s time periods. When monitoring ICP as surveillance of the critically ill patient, it is the score at a certain point of time or during a shorter time period that guides patient management; here that corresponds to the 6-s time periods. Though it can be disputed what may be regarded as major differences in mean ICP, the present findings that the mean ICP differed >5 mm Hg in >20% of the 6-s time windows in 2 out of 5 patients when comparing findings in two solid sensors and in 4 out of 5 patients when comparing scores from one solid and one fluid sensor raises concerns. Since we compared only accepted 6-s time windows, the differences in mean ICP would have been considerably higher if including all 6-s time windows. In the clinical setting, usually the ICP level (mean ICP) is determined without considering the quality of the ICP signal, i.e. whether the ICP signal contains cardiac induced ICP waves or not. However, given the retrospective design of the study, we are unable to assess to which degree patient management and patient outcome were influenced by the differences in the two signals.

With regard to the MWA, the 6-s time window data were presented as percentage of differences >1 or >2 mmHg, mm respectively. These thresholds were chosen as our experience suggests an upper normal threshold value in the MWA of 4–5 mmHg [3,15]. Accordingly, a difference in MWA >1 mmHg could have impact on patient management and patient outcome. It should be noted that MWRT differed between the solid - and the fluid sensor, and particularly between the solid - and the air-pouch sensors (Table 5). When the MWRT was shorter for the fluid sensors this is probably related to the fact that EVDs may be kept open during monitoring, giving shortened MWRT. For the air-pouch sensor, it is related to the transfer function with variable phase.

The combination of findings when comparing scores from two solid sensors with considerable difference in mean ICP but close to identity in the MWAs, are very similar to results we have previously obtained using two solid sensors when monitoring neurosurgical patients [16,17], and thus should reflect reality. Others also previously showed marked differences in level of ICP measured from two different ICP sensors though the ICP waveforms were not compared [10].

### Properties of the ICP sensors

With regard to the quality of the ICP signal, focus has previously been on the long-term-drift of the sensors, their sensitivity to temperature changes, and sensor accuracy comparisons [18-21]. In contrast, the literature has been scarce on specifications of the ICP sensors. Czosnyka et al. [18] reported that the Codman frequency response goes to more than 30 Hz (slew rate −2200 mm Hg/s), and Piper and Miller [8] indicated an even higher cut-off. This frequency response is higher than the frequency contents anticipated in the ICP signal and should provide for good waveform reproduction. According to the manufacturers, the frequency response of the fluid Edward’s sensor is 40 Hz (response goes to > 200 Hz for the transducer alone). With regard to the air-pouch Spiegelberg sensor, its cut-off was reported by Czosnyka et al. [22] to be 4–5 Hz. We presently found it to be even lower (1–2 Hz).

The time-domain method depends on as exact as possible reproduction of the time domain waveform for measuring peak values, peak excursions and latencies. This means that the transfer function between different sensors should be flat in the most important frequency range (up to 4-5x normal heart rate) and the phase as linear as possible. The present comparisons between sensors showed that the phase was linear over the most important frequency range which is a good feature. With regard to the magnitude of the transfer function, it corresponded poorly with the only past statement about the frequency response of the air pouch Spiegelberg sensor known [22]. Based on our analysis, we conclude that the transfer function between the solid Codman sensor and either the fluid Edwards sensor or the air pouch Spiegelberg sensor is quite variable, affecting both the MWA and the MWRT measures. The variation in MWA and MWRT estimates must be caused by variability in the fluid Edward´s and air-pouch Spiegelberg sensors. This may result in either higher or lower values for both the MWAs and MWRTs of these sensors when compared to the reference solid Codman sensor.

### Impact of hospital environment on ICP scores

There are three major factors related to hospital environment that may affect the ICP scores: Human factors, technical issues and technology issues.

Among the human factors, erroneous zeroing of the ICP sensor is of paramount importance. Some solid sensors, such as the Codman and the Camino, can be zeroed only prior to implantation. The Spiegelberg sensor is the only ICP sensor that performs in vivo zeroing, while the Raumedic sensor performs post-implantation electrical zeroing, though not a true in-vivo (atmospheric pressure) check of the catheter-tip sensor. Using a fluid sensor, the zero pressure level depends on the selected zero point relative to the head or the heart; furthermore, with regard to the head there is no consensus whether the zero point should be at the level of the tragus, the eye, vertex, or the frontal bone.

Several technical issues are involved. Sensor damage may occur at any point of time. The sensor may further respond to electrostatic discharges (ESD’s) [6]. Using a fluid system, the sensor is placed at some distance from the patient, requiring that a fluid filled catheter serves as a connection line between the measurement site (the ventricular fluid) and the sensor site. Air bubbles and/or partial/total occlusion of the fluid catheter by blood cloths or brain tissue allows for erroneous pressure reproduction. Mal-positioning of the catheter (outside of the brain fluid) may also give erroneous pressure readings. The physical properties - and the length of the fluid-filled catheter as well as sensor location relative to the patient also influence pressure readings.

Technology issues relate to the properties of the ICP sensor itself. Presently, we showed that the transfer function varied between solid and fluid/air pouch sensors, in particular, the technology of the air pouch sensor made this sensor type less useful for reproduction of pressure waveforms. The Spiegelberg sensor presently used utilizes an air pouch system that lacks the frequency characteristics needed for proper waveform reproductions. On the other hand, the ability of the Spiegelberg sensor to perform true post-implantation zeroing might be one explanation of the relatively smaller differences in mean ICP we observed when using this sensor.

Both human errors and technical issues may affect the static pressure parameter mean ICP. This is related to the current approach of determining the mean ICP as an absolute value relative to the atmospheric pressure (usually referred to as baseline pressure, reference pressure, or zero pressure level). Accordingly, the ICP sensors are zeroed against the atmospheric pressure, and the mean ICP that is revealed on the screen is a pressure value relative to the atmospheric pressure. The present data show that baseline pressure shifts and drifts in deed represent a clinical issue (Table 3; Figure 5,13). The spontaneous sudden shifts in mean ICP thus resemble those we have previously seen in an experimental setting where the ESD’s impact the zero pressure level of ICP sensors were extensive [6]. They are also similar to those and may explain plain the sudden shifts in the mean ICP that can occur during long-term monitoring within the intensive care unit (ICU) [23].

### Quality control during ICP monitoring

Managing patients according to erroneous ICP scores could be fatal; thus the issue of quality control during ICP monitoring is important. Today’s quality control is usually as follows: In situations where an external drain had been placed for CSF drainage, the static intracranial pressure (mean ICP) can be “semi-quantitatively” assessed by measuring the height of the fluid in the fluid-filled catheter. Also, in patients treated with decompressive craniectomy, the ICP may be crudely evaluated by slight compression at the craniectomy site. Quality assessment of the pressure waveform is done by visual inspection of the ICP waveform on the screen of the vital signs monitor or by inspection of the fluid pulsations within a fluid-filled catheter of a CSF drain. However, as mentioned above even partial occlusion of a CSF drain may give erroneous dynamic pressure reproductions. Also, qualitative assessment of the pressure waveform provides minimal information whether the waveform is normal or not.

A quality control beyond what is mentioned above can be obtained by the use of computer software that in an intelligent manner identifies “noise” in the ICP signal, and also automatically identifies the cardiac induced ICP waves [7]. Increasing proportion of “noisy” waves indicates poor signal quality. In such a setting, the sensor system would need the proper ability to retrieve the ICP waveform. Incorporating ICP waveform evaluation in patient management would also identify situations of erroneous static ICP scores [16,17,24]. Based on how the static mean ICP and the dynamic MWA relates, finding of a pathological high mean ICP together with a normal (low) MWA indicates an erroneous static ICP. From this, monitoring the dynamic intracranial pressure parameters ought to be the primary type of intracranial pressure monitoring. Presently, the solid Codman sensor was superior for measuring ICP waveforms when compared with that of the fluid Edward’s sensor and particularly the air-pouch Spiegelberg sensor.

## Conclusions

Simultaneous monitoring of ICP using two solid sensors may show marked differences in static ICP (mean ICP or ICP level) but close to identity in dynamic ICP waveforms. This indicates that shifts in ICP baseline pressure (sensor zero level) occur during ICP monitoring. Trend plots of the ICP parameters confirm that shift in mean ICP (due to spontaneously changed ICP baseline pressure) in deed occur clinically. Solid sensors are superior to fluid – and air pouch sensors when evaluating the dynamic ICP parameters.

## Abbreviations

ICP, Intracranial pressure; MWA, Mean ICP wave amplitude; SW, Single wave.

## Competing interests

SH and WSO report no conflicts of interest. PKE has financial interest in the software company (dPCom A/S) that manufactures the software (Sensometrics® Software), which was used for digital recording of the continuous pressure signals in this study.

## Authors' contributions

All authors contributed to conception and design, acquisition and interpretation of data. SH contributed with determining transfer functions of the different sensors. PKE contributed the bulk of the drafting of the manuscript and SH and WSO contributed with thorough editing of the manuscript. All authors have read and approved the final manuscript.

## Supplementary Material

Additional file 1 **Category A**Animation of measurements of Signals 1 and 2 in PatID 2. The animation shows the simultaneous continuous ICP waveform of Signals 1 (Codman; lower signal) and 2 (Codman; upper signal) in PatID 2. Note that the ICP waveform is identical while the baseline pressure is different. Click here for file

Additional file 2 **Category B.**Animation of measurements of Signals 1 and 2 in PatID 8. The animation shows the simultaneous continuous ICP waveform of Signals 1 (Codman; lower signal) and 2 (Edwards; upper signal) in PatID 8. Note that the ICP waveform is quite similar while the baseline pressure is different Click here for file

Additional file 3 **Category C.**Animation of measurements of Signals 1 and 2 in PatID 11. The animation shows the simultaneous continuous ICP waveform of Signals 1 (Codman; lower signal) and 2 (Spiegelberg; upper signal) in PatID 11. Note that both the ICP waveform and the baseline pressure are different. Click here for file
